# Advax-CpG Adjuvant Provides Antigen Dose-Sparing and Enhanced Immunogenicity for Inactivated Poliomyelitis Virus Vaccines

**DOI:** 10.3390/pathogens10050500

**Published:** 2021-04-21

**Authors:** Yoshikazu Honda-Okubo, Jeremy Baldwin, Nikolai Petrovsky

**Affiliations:** 1 Vaxine Pty Ltd., Flinders Medical Centre, Bedford Park, Adelaide 5042, Australia; yoshikazu.hondaokubo@flinders.edu.au (Y.H.-O.); Jeremy.baldwin@flinders.edu.au (J.B.); 2 Department of Endocrinology, College of Medicine and Public Health, Flinders University, Adelaide 5042, Australia

**Keywords:** poliomyelitis, virus, vaccine, polio, adjuvant, Advax-CpG

## Abstract

Global immunization campaigns have resulted in a major decline in the global incidence of polio cases, with wild-type poliovirus remaining endemic in only two countries. Live oral polio vaccine (OPV) played a role in the reduction in polio case numbers; however, the risk of OPV developing into circulating vaccine-derived poliovirus makes it unsuitable for eradication programs. Trivalent inactivated polio virus (TIPV) vaccines which contain formalin-inactivated antigens produced from virulent types 1, 2 and 3 reference polio strains grown in Vero monkey kidney cells have been advocated as a replacement for OPV; however, TIPVs have weak immunogenicity and multiple boosts are required before peak neutralizing titers are reached. This study examined whether the incorporation of the novel polysaccharide adjuvant, Advax-CpG, could boost the immunogenicity of two TIPV vaccines, (i) a commercially available polio vaccine (IPOL^®^, Sanofi Pasteur) and (ii) a new TIPV formulation developed by Statens Serum Institut (SSI). Mice were immunized intramuscularly based on recommended vaccine dosage schedules and serum antibody titers were followed for 12 months post-immunization. Advax-CpG significantly enhanced the long-term immunogenicity of both TIPV vaccines and had at least a 10-fold antigen dose-sparing effect. An exception was the poor ability of the SSI TIPV to induce serotype type 1 neutralizing antibodies. Immunization with monovalent IPVs suggested that the low type 1 response to TIPV may be due to antigen competition when the type 1 antigen was co-formulated with the type 2 and 3 antigens. This study provides valuable insights into the complexity of the formulation of multivalent polio vaccines and supports the further development of adjuvanted antigen-sparing TIPV vaccines in the fight to eradicate polio.

## 1. Introduction

The global polio vaccine initiative has been extremely successful, with global immunization coverage now including >80% of all children under 5 years of age, resulting in a major decline in cases of poliomyelitis with the very real prospect of eradicating the virus altogether, a feat that has only been achieved previously for smallpox [1]. Current polio vaccines take two forms—live-attenuated oral polio virus (OPV) and inactivated polio virus (IPV) [2]. OPV advantages include low cost, ease of administration and ability to induce both mucosal and humoral immunity. However, OPV has the disadvantages that it is heavily reliant on the cold chain for retention of vaccine potency and OPV has the propensity to revert to wildtype and cause vaccine-derived poliomyelitis [3]. IPV is much safer as it cannot cause poliomyelitis, but its disadvantages include weak immunogenicity and the need for multiple booster injections, together with high manufacturing costs [4,5]. There is therefore a pressing need to develop more immunogenic and cost-effective IPV vaccines to replace OPV vaccines in global polio eradication programs.

IPV vaccine is based on formalin-inactivated antigens produced from virulent types 1, 2 and 3 reference strains grown in Vero monkey kidney cells [6]. IPV vaccine, when injected intramuscularly, induces serum IgG neutralizing antibodies that prevent polio virus from invading motor neurons, thus preventing the neurological consequences of poliomyelitis infection [7]. The first dose of IPV vaccine is usually given at 1–2 months of age, a second dose at 4 months, a third dose at 6–18 months, and a further booster at 4–6 years [8]. In some cases, a fifth immunization may be required during adolescence. Current IPV vaccines induce 90% protection against all three serotypes after two doses, and 99% after three doses [8]. Current IPV vaccines, whilst effective, could be improved in a number of ways. Notably, the level and duration of protective immunity could be enhanced, reducing the number of booster doses required for protection of infants. Increased immunogenicity could also allow antigen dose sparing, thereby reducing its cost. Although IPV vaccines currently do not contain adjuvant, animal studies have shown the feasibility of using various adjuvants including MF59, calcium, aluminum hydroxide, vitamin D and TLR9-active cytosine-phosphodiester-guanosine (CpG)-motif containing oligonucleotides in IPV vaccines [9,10,11,12,13]. However, most of these studies only evaluated adjuvants with the IPV in their monovalent form and not formulated as TIPV, limiting the interpretation of such results. However, these studies do support the potential benefit of adjuvants for enhancing IPV immunogenicity and for antigen dose-sparing.

Advax is a novel polysaccharide-based adjuvant derived from crystalline particles of delta inulin, a natural plant sugar comprised of fructose and glucose units, thereby providing high levels of biocompatibility [14]. As demonstrated in animal challenge studies, Advax adjuvant enhanced humoral and cellular immunity against a broad range of co-administered viral antigens including seasonal and pandemic influenza, Japanese encephalitis, Murray Valley encephalitis, West Nile, hepatitis B, and peste de petit ruminants, amongst many others [14,15,16,17,18,19,20]. When used in the immunization of pregnant mothers and newborns, Advax adjuvant was safe and well tolerated and enhanced immunogenicity despite pregnancy-associated immunosuppression or neonatal immune immaturity [21,22,23]. The immunogenicity of Advax adjuvant was further extended with the addition of TLR9-active CpG oligonucleotides to the formulation to create Advax-CpG adjuvant, with the CpG oligonucleotide helping to drive a strong Th1 vaccine response [24] and acting synergistically with Advax to enhance vaccine immunogenicity [25,26,27,28]. The current study was undertaken to assess the ability of Advax-CpG adjuvant to improve the immunogenicity and provide antigen dose-sparing for two different commercial IPV vaccine candidates.

## 2. Methods

### 2.1. Animals

All procedures were performed in accordance with the Animal Experimentation Guidelines of the National Health and Medical Research Council of Australia and approved by the Flinders University Animal Welfare Committee. Female BALB/c mice, 6 to 8 weeks of age, and female Wistar rats, weighing between 175 and 250 g, bred under specific pathogen-free conditions, were supplied by the Flinders University animal facility. 

### 2.2. Vaccines and Adjuvants

Inactivated viral antigens, namely Brunhilde 977 D-antigen units (DU)/mL (type 1, IP1), MEF-1 1260 DU/mL (type 2, IP2) and Saukett 1860 DU/mL (type 3, IP3) were provided as monovalent bulk antigens as well as together in a pre-formulated trivalent IPV (TIPV containing 327, 70 and 279 DU for IP1, IP2 and IP3, respectively) supplied by Statens Serum Institut (SSI), Copenhagen, Denmark. DUs were determined by a sandwich ELISA. Anti-D-antigen-specific mouse monoclonal antibodies for each type were used as capture antibodies and type-specific neutralizing rabbit antisera were used as the detection antibodies. D-antigen titers of test samples were measured with reference to WHO S-IPV (91/672) D-antigens by parallel line assay [29]. A commercially available TIPV (IPOL^®^, Batch No. H0022-1) was purchased from Sanofi Pasteur Inc. (Swiftwater, PA, USA). Advax and CpG are proprietary adjuvants produced by Vaxine Pty Ltd. (Adelaide, SA, Australia) with a description of Advax adjuvant outlined in Patent number US201161498577P.

### 2.3. Immunization Schedule for Mouse and Rat Studies

An overview of immunization schedule for both mouse and rat studies is provided in Table 1. Vaccine antigens were combined with Advax and/or CpG formulation by simple admixture immediately prior to intramuscular (i.m.) immunization in volumes of 0.1 (mice) or 0.2 mL (rats). Female BALB/c mice (*n* = 3/group) were immunized i.m. into the hind thigh at weeks 0, 3, 7 and 12 with IPOL^®^ vaccine (8 DU IPtype 1, 1.6 DU type IP2 and 6.4 DU type IP3) alone or formulated with Advax (1 mg) with or without CpG (10 µg). IPOL^®^ was concentrated 4-times to reduce the immunization volume for mice (0.1 mL per mouse). Sera were collected at weeks 2, 4, 9, 13, 26, 30, 35 and 44 after the first immunization for measurement of antibody levels and neutralization activity. For studies with the SSI TIPV vaccine, female BALB/c mice (*n* = 9/group) received two i.m. immunizations into the hind thigh 3 weeks apart with SSI TIPV vaccine (10 DU type adjusted by dilution with saline for injection1, 2 DU type 2 and 9 DU type 3) alone or formulated with Advax (1 mg) with or without CpG (10 µg). An addition group was set up to test monovalent forms of the IPV vaccine at three different doses (10, 1 and 0.1 DU, DU adjusted by dilution with saline) alone or formulated with Advax-CpG or CpG adjuvant alone. Sera were collected at weeks 2, 5, 13, 26, 39 and 52 after the first immunization for measurement of antibody levels and neutralization activity. For rat studies with the SSI TIPV vaccine, female Wistar rats (*n* = 3/group) were immunized i.m. twice (0.2 mL per mouse) 3 weeks apart with SSI TIPV (10 DU type IP1, 2 DU type IP2 and 9 DU type IP3, DU adjusted by dilution with saline) alone or formulated with Advax (2 mg) and/or CpG (20 µg). Blood samples were collected at weeks 2 and 4 for measurement of antibodies and neutralization activity.

### 2.4. ELISA for Detection of Anti-IPV Antibodies

IPV-specific antibodies were determined by ELISA. One hundred microliters of a 1:200 dilution of each IPV strain were absorbed overnight at 4 °C to ELISA plates in 0.1 M sodium hydrogen carbonate buffer, pH 9.6. Wells were blocked with 1% BSA/PBS and 100 µL of serum diluted in 1% BSA/PBS (1:1000 for total IgG and IgG1 and antibodies and 1:200 for IgM, IgG2a, IgG2b and IgG3) were added and incubated for 2 h at RT. For murine studies, after washing, HRP-conjugated anti-mouse IgM (BD Bioscience), IgG (Millipore), IgG1 (BD Bioscience), IgG2a (BD Bioscience), IgG2b (BD Bioscience) and IgG3 (BD Bioscience) were added and incubated for 1 h at RT. For rat studies, HRP-Goat anti-Rat IgG (Chemicon, Cat. No. AP136P) was added and incubated for 1 h at RT. After a final wash, plates were incubated with 100 µL of freshly prepared TMB substrate for 10 min and then the reaction stopped by 1 M phosphoric acid and the optical density measured at 450 nm (OD 450 nm) using VersaMax ELISA microplate reader (Molecular Devices) and data analyzed by SoftMax Pro Software.

### 2.5. Neutralization Assays

Serum specimens were shipped to the Centre for Disease Control (CDC) and Prevention PPLB Lab for analysis of neutralizing antibody titers. Prior to testing, sera were randomized in a balanced-block scheme to minimize systematic errors due to microplate and run position. Sera from a given study arm were proportionately distributed among runs and multiple sera from a given subject were tested in the same run. A CDC reference serum was included in multiple positions in each run, as a positive control, and virus titration was included in each run as a validity control. Diluted sera were combined with virus and cells and incubated under standard cell culture conditions for 5 days. The viral cytopathic effect was detected by staining with crystal violet and optical density measured in a plate reader. The test strains employed included Brunhilde serotype 1, MEF-1 serotype 2, and Saukett serotype 3. Serum neutralization titers were calculated using the Kärber method [30] expressed as log2 (titer). Appropriate positive and negative controls and virus back titrations were included in every run for quality control purposes. Sera was tested in triplicate using eight two-fold serial dilutions starting at 1:8 (and up to 1:1024) and tested against the poliovirus strains described above.

### 2.6. Statistical Analysis

GraphPad Prism 4 for Windows was used for drawing graphs and statistical analysis (GraphPad Software, San Diego, CA, USA). Significant differences between experimental and control groups were analyzed by the Mann–Whitney test. Differences were considered statistically significant when the *p*-value was less than 0.05.

## 3. Results

### 3.1. Advax-CpG Improves IPOL^®^ TIPV Vaccine Long-Term

**Figure 1:**
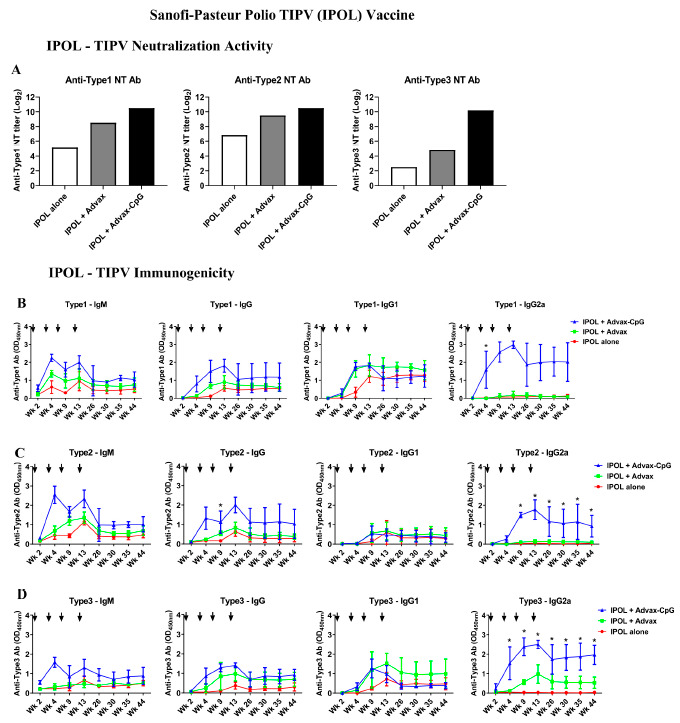
Advax-CpG adjuvant enhances neutralizing activity and long-term immunogenicity of commercial trivalent inactivated polio virus (TIPV) vaccine (IPOL, Sanofi-Pasteur). Female BALB/c mice (*n* = 3/group) received intramuscular (i.m.) immunizations at weeks 0, 3, 7 and 12 with IPOL vaccine (8 DU type 1, 1.6 DU type 2 and 6.4 DU type 3) alone or formulated with Advax or Advax-CpG adjuvants. Sera were collected at weeks 2, 4, 9, 13, 26, 30, 35 and 44 after the first immunization. Neutralization (NT) titers were determined against type 1—Brunhilde, type 2—MEF-1 and type 3—Saukett virus. Due to insufficient sera being available to run NT assays on individual mice, sera from each members of a group were assembled into a single pooled sample to run the NT assay, resulting in a single result for each serotype (**A**). Type-specific antibodies were determined by ELISA and OD values are shown as mean ± SD (**B**–**D**). The significance for ELISA results was analyzed by one-way ANOVA with the Kruskal–Wallis test and was indicated as * *p* < 0.05. Arrows indicate the immunization timing.

As an initial first step, we assessed the ability of Advax-CpG adjuvant to enhance the antibody responses to a commercial human TIPV vaccine, IPOL^®^ (Sanofi, Swiftwater, PA, USA). To mimic the manufacturer’s recommended vaccine schedule in human babies, BALB/c mice were immunized with four doses of IPOL^®^ vaccine at weeks 0, 3, 7 and 12 with IPOL^®^ alone or formulated with Advax or Advax-CpG adjuvant. While ELISAs could be performed on individual mice samples, due to larger sera requirements for the neutralization titer (NT) assays, these were unable to be performed on individual mice, with sera from each member of a group being assembled into a single pooled sample to run the NT assay. Overall, unadjuvanted IPOL^®^ vaccine had low immunogenicity after administration of two consecutive 0.1 mL doses, with NT on the pooled group sera of 36, 113.7, and 5.6 for IPV1, IPV2 and IPV3, respectively (Figure 1A). By contrast, when the IPOL^®^ vaccine was formulated with Advax-CpG adjuvant, the NT for each of the three IPV antigens were markedly boosted in the pooled sera from each group, achieving NT > 1:1024 against each of the three IPV strains (Figure 1A). While the use of pooled group samples precluded the use of statistics to compare groups, the 10–200-fold higher NT for each serotype in the adjuvanted groups clearly demonstrated a major effect of the adjuvants, and this was further supported by the ELISA data on individual mice. We then boosted the mice with two additional higher doses of the IPOL^®^ vaccine (triple-dose-0.3 mL equivalent) at weeks 12 and 14 to see whether this could boost the low titers induced by the IPOL^®^ vaccine alone and then followed the resulting antibody titers for a total of 52 weeks. Notably, even after a total of four doses including two triple-dose boosters, the commercial IPOL^®^ vaccine was, by itself, unable to match titers achieved after just two standard doses of IPOL^®^ combined with Advax or Advax-CpG adjuvants. IPV3 neutralization antibody titers, in particular, remained significantly low even after four doses of unadjuvanted IPOL^®^ vaccine. By contrast, high neutralization titers to IPV1, IPV2 and IPV3 were achieved in the Advax and Advax-CpG adjuvated vaccine groups with titers remaining above levels predicted to be protective (titer of 1:8) all the way to study termination at 52 weeks post-immunization.

### 3.2. Advax-CpG Induces a Strong Th1 Immune Response to IPOL^®^ TIPV Vaccine 

The addition of Advax-CpG adjuvant to the IPOL^®^ TIPV vaccine enhanced not only the neutralizing antibody response but also modulated cellular Th1/Th2 balance. Unadjuvanted TIPV vaccine induced a typical picture with a predominant IgG1 response to all three IPV strains, consistent with an overall Th2 bias (Figure 1B). When formulated with Advax adjuvant, there was enhancement of both IgG1 and IgG2a responses (Figure 1C). By contrast, formulation with Advax-CpG adjuvant resulted in a persistent IgM response together with a strong IgG2a response, consistent with a Th1-bias (Figure 1B).

### 3.3. Dose-Sparing Effects of Advax-CpG Adjuvant on SSI TIPV Immunogenicity

**Figure 2:**
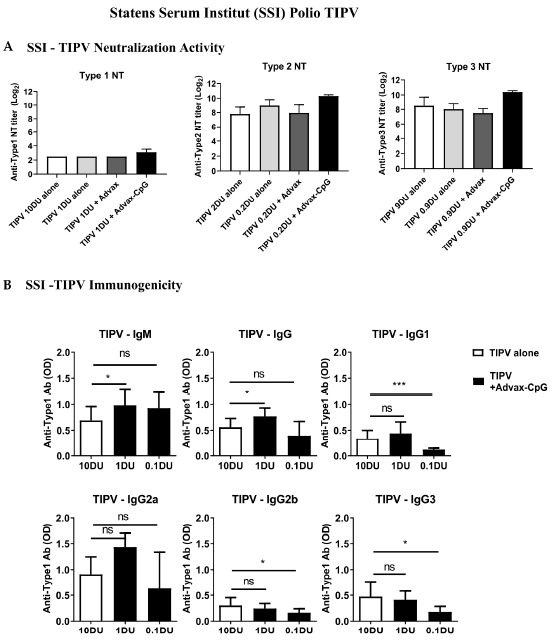
Dose-sparing effect of Advax and Advax-CpG adjuvant on an experimental TIPV vaccine (SSI). Female BALB/c mice (*n* = 9/group) received two i.m. immunizations 3 weeks apart with indicated dose of TIPV vaccine with (Grey and Black bars) or without (White bars) Advax or Advax-CpG. Serum neutralizing (NT) titers against type 1—Brunhilde, type 2—MEF-1 and type 3—Saukett were calculated using the Kärber method (Mean + SD). (**A**). Dose-sparing effect of Advax adjuvant for serotype 1 was further determined by ELISA (**B**). Antibody values are mean + SD with significance indicated as * *p* < 0.05, *** *p* < 0.001 or NS; not-significant compared with 10 DU antigen alone group using Mann–Whitney test.

We next evaluated another TIPV under development by SSI. BALB/c mice were immunized with two doses of SSI TIPV vaccine 3 weeks apart formulated with or without Advax or Advax-CpG adjuvants. The antigens were mixed in the same ratios as in human TIPV vaccine, i.e., 327, 70 and 279 DU of IPV1, IPV2 and IPV3, respectively. The SSI TIPV induced high type 2 and 3 neutralizing antibody titers, with Advax-CpG further boosting this response (Figure 2B). Notably, even a 1/10th dose of SSI TIPV formulated with Advax-CpG achieved higher titers than a full dose of TIPV vaccine alone. The TIPV vaccine induced only modest neutralizing antibody titers to type 1 that after 2 doses remained below protective levels even with an IPV1 dose of 10 DU, with the addition of Advax-CpG not significantly improving titers to IPV1 (Figure 2B). The neutralizing antibody results were surprising as Advax-CpG significantly boosted IPV1 antibody levels by ELISA and have an antigen dose-sparing effect with 1/10th dose of TIPV (1 DU) formulated with Advax-CpG achieving the same level of Type 1 IgG1, IgG2b and IgG3 as TIPV alone at 10 DU (Figure 2B).

### 3.4. Effects of Advax Adjuvants on Antibody Kinetics and Longevity

**Figure 3:**
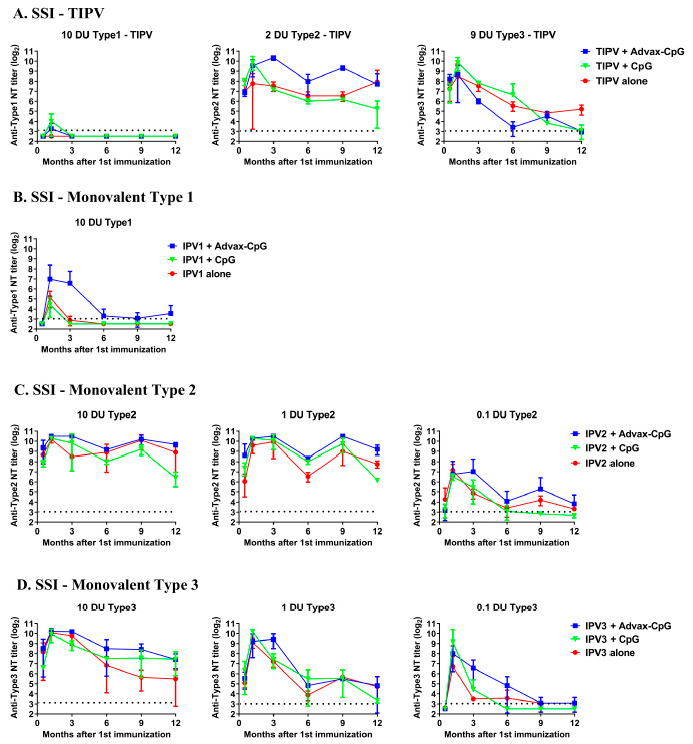
Kinetics and longevity of SSI trivalent and monovalent IPV neutralization antibodies. Female BALB/c mice (*n* = 9/group) received two intramuscular immunizations 3 weeks apart with indicated dose of trivalent (**A**) or monovalent (**B**–**D**) IPV vaccine alone (red) or formulated with Advax-CpG (blue) or CpG (green). Sera collected at indicated time-points were analyzed by virus neutralization assay against type1—Brunhilde, type 2—MEF-1 or type 3—Saukett virus. Serum neutralizing titers were calculated using the Kärber method (Mean ± SD). Dotted line placed at minimum protection level. SSI—Monovalent Type 1 at antigen dose of 0.1 and 1 DU not shown as they were below detection limit.

To determine whether antigenic competition may be responsible for the lack of a type 1 neutralizing antibody response to the SSI TIPV vaccine, BALB/c mice were immunized with two doses 3 weeks apart of IPV monovalent at 10, 1 and 0.1 DU formulated with or without Advax-CpG or CpG alone and followed over 12 months. A group immunized with SSI TIPV vaccine was used as a control. Interestingly, the type 1 IPV monovalent vaccine (10 DU) induced neutralization titers (Figure 3B) significantly higher TIPV vaccine containing the same dose of type 1 (10 DU) (Figure 3A). The type 1 IPV monovalent (10 DU) formulated with Advax-CpG maintained IP1 neutralization activity above protective levels for the course of the 12 months of follow up (Figure 3B). The type 1 IPV monovalent at 1 or 0.1 DU failed to induce type 1 neutralization titers above protective levels (data not shown). All type 2 and 3 IPV monovalent vaccine formulations induced high type 2 and 3 neutralization titers that remained above protective levels in groups immunized with either 1 and 10 DU (Figure 3C,D). At an antigen dose of 0.1 DU, only the Advax-CpG adjuvanted IPV monovalent groups maintained type 2 and 3 neutralization titers above protective levels throughout the 12 months of the study, in comparison to animals immunized with IPV monovalent where neutralizing antibody titers fell below protective levels approximately 6 months after immunization (Figure 3C,D).

**Figure 4:**
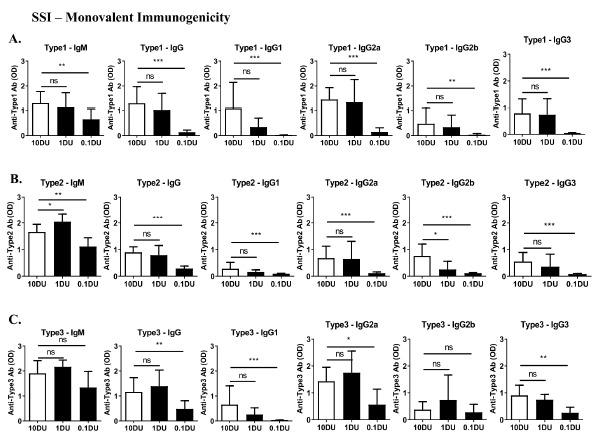
Dose-sparing effect of Advax-CpG adjuvant on SSI Monovalent IPV measured by ELISA and virus neutralization assay. Female BALB/c mice (*n* = 9/group) received two intramuscular (i.m.) immunizations 3 weeks apart with 10 DU of monovalent IPV vaccine alone (white bars) or 1 DU or 0.1 DU IPV antigens with Advax-CpG adjuvant (black bars). Serum collected 2 weeks after the second immunization was analyzed by ELISA against each vaccine antigen: type 1—Brunhilde (**A**), type 2—MEF-1 (**B**) and type 3—Saukett (**C**). Antibody values are mean + SD with significance indicated as * *p* < 0.05, ** *p* < 0.01, *** *p* < 0.001 or NS; not-significant compared with 10 DU IPV vaccine alone group using the Mann–Whitney test.

Serum antibody responses were measured by ELISA 2 weeks after the second immunization. A 1 DU dose of IPV monovalent antigens formulated with Advax-CpG adjuvant induced similar antibody levels, with the exception of type 2 -IgG2b, as 10 DU of IPV antigen alone, consistent with ten-fold antigen sparing (Figure 4A–C). Similar to what was seen with the IPOL vaccine, the addition of Advax-CpG adjuvant had a marked effect on broadening the antibody response to the SSI TIPV vaccine from a IgG1predominant response to one also including IgG2a, IgG2b and IgG3.

### 3.5. TIPV Vaccine Formulated with Advax-CpG Adjuvant Induces Protective Antibody Levels in Rats

**Figure 5:**
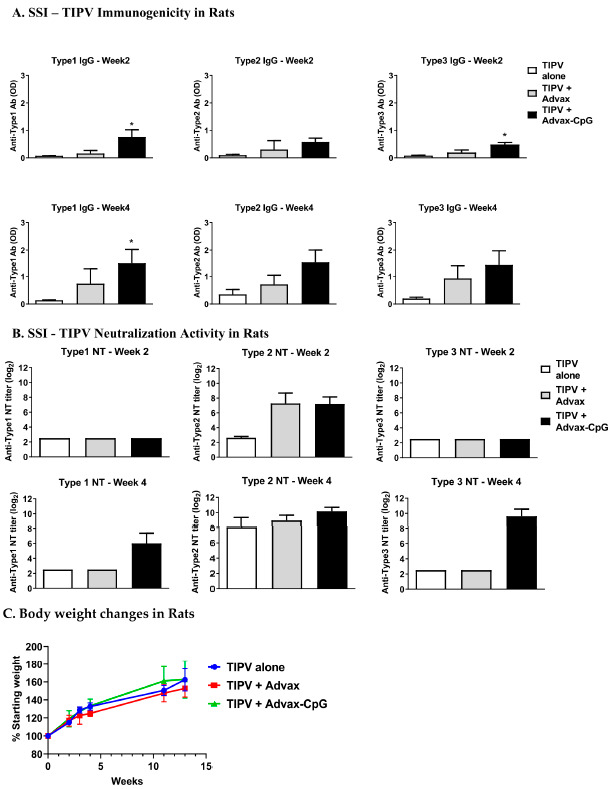
Neutralization activity and immunogenicity of SSI trivalent inactivated polio virus vaccine in rats. Female Wistar rats weighing between 175 and 250 g, were immunized i.m. twice 3 weeks apart with SSI TIPV (type 1—10 U, type 2—2 U, type 3—9 DU) alone or formulated with Advax or Advax-CpG. Blood samples were collected at week 2 and 4 for measurement of total IgG by ELISA (**A**) and neutralization activity (**B**). Antibody values are mean + SD. Serum neutralizing was calculated using the Kärber method (Mean + SD). Significance was analyzed by one-way ANOVA with the Kruskal–Wallis test and was indicated as * *p* < 0.05. Body weights were recorded until 13 weeks after the first immunization (**C**).

To evaluate whether host species played a role in the lack of immune response to type 1 antigen in the SSI TIPV vaccine, female Wistar rats were immunized i.m. twice 3 weeks apart with SSI TIPV and blood was collected at 2 weeks (1 week post-first immunization) and 4 weeks (1 week post-boost) to measure antibody levels and neutralization activity. Similar to the murine studies, SSI TIPV formulated with Advax-CpG adjuvant enhanced the levels of IgG antibodies to TIPV in rats (Figure 5A). SSI TIPV alone induced only weak neutralization titers to type 1. However, unlike the results of murine studies, SSI TIPV formulated with Advax-CpG adjuvant induced a high type 1 neutralization titer (GMT of ~64) in rats (Figure 5B). Type 2 neutralization titers for SSI TIPV formulated with Advax-CpG were protective after only one immunization (GMT = ~150) and were further enhanced by a booster immunization (GMT = 1157) (Figure 5B). Type 3 neutralizing titers for SSI TIPV formulated with Advax-CpG reached protective levels after the second dose with a GMT of 789, whilst titers for SSI TIPV alone remained low at 4 weeks with a GMT of ~5–6 (Figure 5B).

## 4. Discussion

With the global eradication of polio in sight, there is an urgent need to develop immunogenic and cost-effective TIPV vaccines to provide coverage in the last remaining endemic regions. This study confirmed the ability of Advax-CpG adjuvant to enhance IPV immunogenicity and provide dose-sparing in two IPV vaccines; a commercially available TIPV vaccine from Sanofi Pasteur Inc, IPOL^®^, and a new TIPV vaccine under development by SSI.

Advax-CpG when added to IPOL^®^ TIPV induced higher neutralization titers against each of the three polio strains. Advax adjuvant enhanced the durability of immune response, with protective levels of neutralizing antibodies lasting to 1-year post-immunization. Enhancement of serum antibody levels was associated with an increased frequency of memory B cells in immunized animals.

Whilst both Advax and Advax-CpG adjuvant provided enhanced IPV immunogenicity, Advax-CpG provided slightly higher antibody titers and induced a switch from IgG1 to IgG2a, indicative of a change from T helper 2 (Th2) to T helper 1 (Th1) immune response. Th1 cells produce proinflammatory cytokines, such as INF-γ, that enhance cellular immune response effective against intracellular pathogens, such as polio virus, whilst Th2 cells predominately produce cytokines, such as IL-4, that boost antibody production [31]. Antibody subclasses are governed by the balance of cytokines produced by Th1 and Th2 cells, with IL-4 promoting the production of IgG1, whilst IFN-γ enhances the production of IgG2 subtypes [32]. The Advax-CpG-driven Th1 response, as reflected by high IgG2 production, is consistent with the literature in showing that CpG oligonucleotides induce Th1 immunity [24]. This Th1 bias of Advax-CpG adjuvant may help to reduce the risk of vaccine allergic sensitization. For example, Advax-CpG adjuvant prevented eosinophilic lung immunopathology caused by inactivated SARS coronavirus vaccine [27]. Yang et al. [13] demonstrated that CpG formulated with alum enhanced the immunogenicity and provided 4–16-fold dose sparing for a Sabin poliovirus vaccine although, a major limitation was that the IPV antigens were only evaluated in monovalent form. While other studies have reported that CpG adjuvants can cause local reactions [33], no local or systemic reactogenicity was observed in the immunized mice or rats in the current study. The lack of reactogenicity or pyrogenicity in the Advax-CpG may be attributed to the unique anti-inflammatory properties of Advax [14].

Advax-CpG, when added to SSI TIPV, conferred at least 10-fold antigen dose sparing. Unlike the IPOL^®^ TIPV, the SSI TIPV vaccine appeared to have a major inbuilt Th1 bias, as reflected by predominant IgG2a/b antibody induction even when administered without adjuvant. While protective levels of type 2 and 3 neutralization activity persisted long-term (>1 year), type 1 neutralization titers remained below protective levels. Similar results were seen in rats immunized with SSI TIPV, although in that case the low type 1 neutralization titer was overcome to an extent by the addition of Advax-CpG adjuvant. Immunization of mice with type 1 monovalent antigen formulated with Advax-CpG enhanced neutralization titers compared to the same antigen dose in the TIPV formulation. Thus, these data suggest that when the three SSI antigens are formulated together as TIPV, there might be an antigenic competition which then preferences B cell responses to type 2 and 3 antigens over type 1. Antigenic competition is not an uncommon problem in multivalent vaccines, particularly where the antigens are closely related. A previous study evaluating the SSI TIPV vaccine similarly reported lower type 1 titers neutralization in microneutralization assays run by the Centre for Disease Control laboratory when compared to type 2 and 3 run in their inhouse assays, suggesting possible differences in assay protocols or antigens [34]. As Advax-CpG had a dose-sparing effect on type 2 and 3 antigens inducing very high antibody titers to these antigens, future studies could examine whether reducing the dose of type 2 and 3 antigens in the TIPV formulation could overcome type 1 antigen competition.

This study also highlighted the complexity of IPV antigens, with different effects of IPV antigens from SSI and Sanofi Pasteur. In particular, the SSI IPV antigens appeared to have a major inbuilt Th1 bias, as reflected by predominant IgG2a/b antibody response even when the antigens were administered without adjuvant. By contrast, the Sanofi commercial TIPV vaccine induced almost exclusively IgG1 antibodies when given without adjuvant. One explanation for SSI antigens to have a high Th1 bias would be if it contained residual nucleic acids after inactivation which are thereby able to bind to TLRs and induce innate immune activation, thereby behaving like an inbuilt adjuvant. This was also seen in the recent Dietrich et al. [34] paper where in fact they saw no IgG1 production in response to the TIPV antigen either alone or combined with CAF01 adjuvant, but instead only detected IgG2a/c and IgG2b, suggesting an Th1-bias of the IPV antigens. Such an intrinsic adjuvant effect could mask the full benefits of added adjuvants. Whilst some residual nucleic acids may assist a vaccine by acting as adjuvants, they could also be a major cause of reactogenicity. For example, an Australian pediatric seasonal influenza vaccine had to be withdrawn from the market after causing febrile convulsions in children, a problem identified to be due to unusually high levels of residual nucleic acids due to inadequate splitting of the beta-propiolactone inactivated virus particles [35,36]. IPV, like seasonal influenza vaccine, is based on an inactivated RNA virus and hence high Th1 activity of IPV antigens may predict excess reactogenicity and potential to cause fevers in very young children, something that will need to be carefully monitored.

Extensive studies are ongoing into the mechanism of action of Advax and Advax-CpG adjuvants. The results to date indicate that antigen absorption and depot formation is not required for Advax adjuvant action, with an adjuvant effect observed even when Advax was injected 24 h in advance of the antigen, but not when injected 24 h after the antigen [19]. An extensive monocytic cell infiltrate is observed at the muscle site within 24–48 h of Advax injection indicating that it has major chemotactic effects, and recruited mononuclear cells exhibit upregulation of co-stimulatory molecules including major histocompatibility molecules, CD11c and CD86, but not inflammatory cytokine production, suggesting its major action is on regulation of antigen presenting cell function. Human subjects immunized with Advax-adjuvanted trivalent inactivated influenza vaccine demonstrated an enhanced day 7 post-immunization plasmablast response, with the plasmablasts showing greater frequency of non-silent mutations in the B-cell-receptor CDR3 sequence together with higher levels of activation-induced cytidine deaminase [37]. This supports the idea that Advax adjuvant enhances B cell affinity maturation, which we hypothesize is via an effect to enhance the action of T follicular helper T cells. Hence, delta inulin-based adjuvants particularly when co-formulated with a TLR9 active CpG oligonucleotide potently enhance vaccine immunogenicity and may be useful for antigen-sparing for TIPV vaccines being used in global eradication campaigns.

As IPV vaccine is typically given to young infants, vaccine safety and tolerability are of paramount importance. Reassuringly, IPV formulated with either Advax or Advax-CpG adjuvant did not induce any measurable local or systemic reactogenicity in the immunized mice or rats (data not shown). This is consistent with the strong safety profile of these adjuvants observed in other vaccine studies, including when administered to either pregnant mothers or newborns [21,22,23]. Whilst human pediatric studies have yet to be undertaken, many adult human studies have been conducted of Advax and Advax-CpG in vaccines against influenza, hepatitis B, insect sting allergy and SARS-CoV-2, together involving over 2000 human exposures [16,17,38,39]. These studies confirmed the safety and tolerability of these adjuvants. Altogether, the results indicate that Advax-CpG is a potent adjuvant that is capable of enhancing the immunogenicity of TIPV vaccines and warranting further studies and human trials.

## Figures and Tables

**Table 1:** Experimental Design for Sanofi-Pasteur and Statens Serum Institut (SSI) Polio Vaccine Studies.

**Sanofi-Pasteur (IPOL) Polio Vaccine**					
**Study No.**	**Animals**	**Vaccine Antigen**	**Adjuvant**	**Dose Schedule**	**Purpose of Study**
1	Female BALB/c	Trivalent inactivated polio (8 DU type 1, 1.6 DU type 2 and 6.4 DU type 3)	Alone or with Advax or Advax-CpG	4 doses (weeks 0, 3, 7 and 12)	Long term immunogenicity/Neutralization Activity
**Statens Serum Institut (SSI) Polio Vaccine**					
**Study No.**	**Animals**	**Vaccine Formulation**	**Adjuvant**	**Dose Schedule**	**Purpose of Study**
2	Female BALB/c	Trivalent inactivated polio (10 DU type 1, 1 DU type 2 and 9 DU type 3) at 1, 1:10 and 1:100 dose	Alone or with Advax or Advax-CpG	2-doses (3 weeks apart)	Immunogenicity/Neutralization Activity/Antigen dose sparing capacity
3	Female BALB/c	Trivalent inactivated polio and Monovalent at three different doses at 1, 1:10 and 1:100 dose	Alone or with Advax-CpG or CpG	2-doses (3 weeks apart)	Antigen competition/Long-term Immunogencity
4	Female Wistar rats	Trivalent inactivated polio (10 DU type 1, 1 DU type 2 and 9 DU type 3) at 1, 1:10 and 1:100 dose	Alone or with Advax or Advax-CpG	2-doses (3 weeks apart)	Species-specific differences Immunogenicity/Neutralization Activity/Weight loss

## Data Availability

The data presented in this study are available on request from the corresponding author.
